# A Novel Cross-Layer Routing Protocol Based on Network Coding for Underwater Sensor Networks

**DOI:** 10.3390/s17081821

**Published:** 2017-08-08

**Authors:** Hao Wang, Shilian Wang, Renfei Bu, Eryang Zhang

**Affiliations:** College of Electronic Science and Engineering, National University of Defense Technology, Changsha 410000, China; wanghao08@nudt.edu.cn (H.W.); borenfei15@nudt.edu.cn (R.B.); zhangeryangnudt@163.com (E.Z.)

**Keywords:** underwater sensor networks, network coding, routing protocol, cross-layer design

## Abstract

Underwater wireless sensor networks (UWSNs) have attracted increasing attention in recent years because of their numerous applications in ocean monitoring, resource discovery and tactical surveillance. However, the design of reliable and efficient transmission and routing protocols is a challenge due to the low acoustic propagation speed and complex channel environment in UWSNs. In this paper, we propose a novel cross-layer routing protocol based on network coding (NCRP) for UWSNs, which utilizes network coding and cross-layer design to greedily forward data packets to sink nodes efficiently. The proposed NCRP takes full advantages of multicast transmission and decode packets jointly with encoded packets received from multiple potential nodes in the entire network. The transmission power is optimized in our design to extend the life cycle of the network. Moreover, we design a real-time routing maintenance protocol to update the route when detecting inefficient relay nodes. Substantial simulations in underwater environment by Network Simulator 3 (NS-3) show that NCRP significantly improves the network performance in terms of energy consumption, end-to-end delay and packet delivery ratio compared with other routing protocols for UWSNs.

## 1. Introduction

Underwater wireless sensor networks (UWSNs) have attracted much attention in recent years due to the wide application of marine mining and exploitation [1,2,3,4,5,6]. However, the adverse characteristics of UWSNs raise challenge to the design of reliable data transmission protocols. One of the outstanding prerequisites to establish underwater ad-hoc networks is to find a reliable path to forward data packets to sink nodes. So the design of underwater routing protocols is a key issue in UWSNs applications. A proper routing protocol can significantly reduce the transmission delay, decrease the energy consumption and improve the stability of system. However, there are several unfavorable factors that limit the design of underwater routing protocol. The optical waves and radio waves do not propagate well in underwater environment [7,8]. So most UWSNs applications choose sound waves for data transmissions. The average propagation speed of sound waves in underwater environment is only 1500 m/s, which is much lower than that of optical waves and radio waves (about $3\times 10^{8}$ m/s). So the propagation delay and round-trip time (RTT) in UWSNs are much longer than that in terrestrial applications. Besides, the underwater channel is relatively complex as carrier frequencies of sound waves often share the same frequency band with noise frequencies caused by marine organisms, shipping activities, sea winds and so on. Meanwhile, sound rays are easily affected by the angle of incidence, reflections, sea water temperature, salinity and so on, which will cause severe multipath effects. Moreover, the nodes placed in sea water often move passively with water currents and some communication devices can move at a high speed (like submarine). These movements can result in severe doppler distortions. So underwater data transmission will suffer serious interference by these factors and packet error rate (PER) is extremely high especially when we need long-distance communications. What is worse, the energy consumption of nodes for underwater applications is relatively high due to the difficulties of recharging batteries and the usage of acoustic communication [9]. So we must consider these factors when designing the routing protocols for UWSNs.

Current terrestrial wireless routing protocols often require the acquisition of nodes information in the entire network and this may raise the demands of instant information interaction and frequent feedbacks. However, the long propagation delay and high energy consumption in UWSNs limit the utilization of such protocols. Based on the adverse characteristics of UWSNs, many researchers make great contributions to the exploration of underwater routing protocols. Geographic routing [10,11,12], also called position-based routing, has been proposed recently as a proper solution for UWSNs routing protocol. It tempers the demands for global establishment of complete routes, and the nodes in network can make routing decisions locally. The abundant research of underwater positioning techniques also stimulates the development of geographic routing. To further overcome the void area problem in UWSNs, opportunistic routing [9,13] is introduced into UWSNs routing design. In opportunistic routing, packets forwarding is enhanced by taking advantage of the multiple nodes reception and forwarding. However, the combination of geographic routing and opportunistic routing often causes the redundant packet transmission problem and decreases the channel utilization. Besides, the void area problem still exists as many current UWSNs routing protocols cannot fully use all the receiving nodes in the network and many alternative paths are suppressed to prevent duplicated packet transmissions. Furthermore, the network coding technique [14,15,16] has been proposed for data transmissions in underwater environment for several years and has been verified to be a promising technique for reliable data transmission in UWSNs. However, the network coding technique cannot been applied in practical UWSNs because too many nodes are involved in data transmissions and there are no corresponding routing protocols for this technique. In this paper, we propose a novel cross-layer routing protocol base on network coding (NCRP) for UWSNs to overcome the drawbacks of current UWSNs routing protocols. NCRP is a kind of sender-side routing protocol but it does not need to keep updating neighborhood information frequently as most sender-side protocols do. It combines the design of transport layer and network layer. The transport protocol we use here is network coding based hybrid ARQ protocol (NCHARQ) [16]. The maintenance and correction of paths are operated along with data packets transmissions and thus our proposed protocol can increase channel utilization rate and reduce energy consumption significantly. The main contributions of this paper are listed as follows:

1. Extended opportunistic receiving mode. We design a routing protocol with a primary path and several secondary nodes. The nodes in the network can not only receive packets from their previous hop nodes in the primary path, but also obtain encoded packets from long-distance nodes under certain probabilities. This design can make full use of multicast nature in underwater transmissions and save energy. The network is more robust to environment changes as multiple nodes can participate in one decoding procedure and the breakdown of a single node will not effect the network performance heavily.
2. Based on the node mobility in underwater environment, we design a new route maintenance and update algorithm. The new algorithm can delete or add nodes when detecting inefficient transmissions without frequently updating neighborhood information with beacon messages. So packet collisions can be reduced and the route breakdown can be avoided. Moreover, data transmissions can be more efficient and thus energy consumption is decreased.

The rest of this paper is organized as follows. In Section 2, we review the related works in this field. Next, we give the design background in Section 3, which includes our channel model, network model and an overview of NCHARQ. We present the detailed design of NCRP in Section 4. The effect of some parameter settings and simulation results are given in Section 5 and we conclude our paper in Section 6.

## 2. Related Works

In this section, we review some of the typical routing protocols in UWSNs. To clarify the advantages and disadvantages of different routing protocols clearly, we classify current routing protocols into sender-side, receiver-side and hybrid protocols based on candidate set selection nodes.

In sender-side candidate set selection procedures, the selection of next-hop nodes is decided by senders. Generally, destination nodes need to broadcast beacon signals periodically to ensure that every sending node in network can acquire the information of its neighbor nodes. Based on the neighbor information stored in sending nodes, a cluster of candidate nodes are chosen and sorted according to their priorities. The header of data packets should include the unique IDs of the chosen nodes. Once a node receives a data packet, it checks if it is in the chosen candidate set. Nodes in the chosen set then set a timer to forward the packet to the destination. Those nodes with higher priorities will send packets at an early time and therefore suppress the sending procedure of other nodes with lower priorities. Some typical underwater routing protocols in this category are hydraulic pressure based anycast routing (HydroCast) [17], void-aware pressure routing (VAPR) [18], geographic and opportunistic routing (GEDAR) [13] and pressure sensor based reliable routing (PSBR) [7]. In HydroCast, each node maintains a list of Expected Packet Advance (EPA) of its neighbors. When a node determines to send packets, it performs the opportunistic routing paradigm and the next-hop node priority is set according to EPA. A dead end recovery method is also proposed to search for new paths when there exists void areas. VAPR is similar to HydroCast but uses sequence number, hop count and depth information embedded in periodic beacons to set up next-hop direction and to build a directional trail to the closest sonobuoy. GEDAR improves VAPR by performing the depth adjustment of the void nodes. When the channel senses a void area, GEDAR can move void nodes to new depths to resume the geographic routing. PSBR adopts link quality estimator along with depth distance and residual energy to select a single reliable node for data forwarding. Protocols that fall into this category can choose a subset of candidate forwarding node in advance and therefore more complex and comprehensive fitness functions can be used to choose next-hop nodes. However, due to the node mobility in underwater environment, sending nodes need to keep updating neighborhood information frequently by using beacon messages, which results in high packet collisions and low channel utilization rate.

For receiver-side candidate set selection procedures, the selection of next-hop forwarding nodes is processed by data packets receivers. Sending nodes do not store any information of their neighborhood nodes. At the beginning of sending procedure, sending nodes add some control information (e.g., its depth or position) into packet header. All neighborhood nodes determine locally whether they are responsible for forwarding the packets and set their priorities based on some principles (e.g., residual energy or distance to the sender) after removing the packet header. If a node determines to forward the packet to the destinations, it sets a timer before sending the packet according to its priority and the sending procedure will be cancelled if a duplicated packet is received in this period of time. Protocols that fall into this category are vector-based forwarding protocol (VBF) [11] and its improved version hop-by-hop vector-based forwarding (HHVBF) [12], depth-based routing (DBR) [10], weighting depth and forwarding area division DBR routing (WDFAD-DBR) [19] and so on. VBF uses the 3D geographic position of nodes to build a virtual pipe from the source node to the destination node. The nodes in the network have higher priorities to forward data packets if they are closer to this pipe and the destination node. HHVBF improves VBF by relaxing the restriction of the single pipe line. In HHVBF, each node can be treated as the source node when it needs to forward data packets and a series of pipe lines can be built to search for more alternative routes towards the destination. DBR protocol only uses the depth information for routing process. The nodes in the network just forward data packets to the nodes with lower depth. This operation decreases the difficulty of acquiring 3D geographic position and is especially suitable for applications with multiple sinks. WDFAD-DBR improves DBR by selecting the next forwarding nodes according to the weighting sum of depth difference of two hops. So the probability of meeting void areas can be reduced. Meanwhile, a mechanism of neighbor node prediction is designed to improve the transmission efficiency. Compared with sender-side candidate set selection procedures, these protocols do not need to update neighborhood information by using beacon messages, thus energy consumption is reduced and channel utilization rate is increased. However, these protocol cannot eliminate duplicated transmissions completely. This adverse effect is particularly obvious when considering high PER and node mobility in underwater environment. Besides, when there is a void area in network, these protocols cannot sense the void region in advance and have to increase redundant transmissions to improve packet delivery ratio. Meanwhile, in these protocols, all nodes need to obtain the real-time position of destination nodes or destination nodes need to be densely distributed on water surface. This means any position change of destination nodes needs to be broadcast to the whole network, or we must allocate enough destination nodes on water surface. So the use of such protocols is limited in UWSNs.

The hybrid protocols are relatively complex compared with sender-side and receiver-side protocols. There are three steps in the hybrid protocols: (1) Sender nodes broadcast request packets into the network. (2) Neighborhood nodes of the sender determine if they are responsible for forwarding the packet according to the request information in the packet. Then the chosen nodes will send a reply to the sender. (3) The sender then choose appropriate nodes according to these replies and send data packets to the chosen node. Some typical underwater routing hybrid protocols in this category are focused beam routing (FBR) [20] and channel-aware routing (CARP) [21]. In FBR, nodes know their own location and the location of their final destination. When a node determine to send a packet, it sends a control packet first and waits for replies from its neighbors. The neighbors in a cone centered on the line between the sender and the sink reply with their position along with other information. The neighbor node with the smallest distance towards the sink is chosen as the next hop forwarder. So the selection of the next relay is made at each step of the path. CARP improves FBR by considering link quality for selecting the next-hop relay. The history of successful transmissions to neighbor nodes is maintained in the senders and the relay selection is based on hop count, link quality, buffer space and residual energy. Advantages and disadvantages of this kind of protocols are obvious. The beacon messages do not need to be updated periodically and the sender can obtain its neighborhood information before sending packets. However, the propagation delay of the three steps procedure can increase the communication delay to a great extent and thus this kind of protocols can only be used in low communication rate applications.

To overcome the high packet error rate and unstable channel quality in UWSNs, network coding is proposed and introduced into underwater data transmissions. In [14], the authors apply network coding to UWSNs for reliable data transfer and demonstrate the efficiency of the scheme. The performance can be improved by reducing the paths as in [15], where a twin path and network coding (TPNC) protocol is proposed to achieve lower energy consumption. Although network coding can improve the transmission reliability greatly, it also brings heavy redundant transmission problem. In [16], the authors propose a network coding based hybrid ARQ protocol (NCHARQ) which combines network coding and hybrid automatic repeat request (ARQ) techniques in underwater data transmissions. This protocol reduces the redundant transmissions significantly with the help of channel quality feedbacks and transmission rate control. However, these protocols mainly deal with transmission problems in transport layer, which do not consider the routing protocol in network layer. The relays are supposed to be chosen by a perfect method beforehand and this assumption restricts the practical application of network coding in UWSNs.

## 3. Background

The design of UWSNs routing protocol must be on the basis of its unique characteristics of underwater environment. So we present the channel model and the network model in this part. Moreover, as we need to design routing protocol and transport protocol jointly, we also present a review of NCHARQ in this section.

### 3.1. Channel Model

We give an algorithm of calculating packet error rate (PER) for two neighborhood nodes in underwater channel here. The main factors that affect the transmission performance of underwater channel are propagation loss, ambient noise and fading. For propagation loss, Urick gave an empirical formula in 1967 [22] as shown in Equation (1).

(1) $$TL\left(d,f\right)=A_{0}d^{k}a{\left(f\right)}^{d}$$

Here $A_{0}$ is a normalizing parameter. *d* is the transmission range. *k* is the spread factor and k=1.5 is usually taken as representative of practical spreading based on a partially bounded sphere for shallow sea channel. The sound wave absorption loss $a\left(f\right)$ is modeled by the famous Thorps formula [23] in Equation (2)

(2) $$10\log a\left(f\right)=\left\{\begin{array}{cc} 0.11\frac{f^{2}}{1+f^{2}}+44\frac{f^{2}}{4100+f^{2}}+2.75\cdot 10^{-4}f^{2}+0.003, & f>200\mathrm{Hz}\\ 0.002+0.11\frac{f^{2}}{1+f^{2}}+0.11f^{2}, & f<200\mathrm{Hz} \end{array}\right.$$

The ambient noise in underwater channel is mainly affected by four factors, including turbulence $N_{t}$, shipping $N_{s}$, waves $N_{w}$ and thermal noise $N_{th}$. According to the famous Wenz noise power spectral density [24], the effect of ambient noise can be described as in Equations (3) and (4):

(3) $$NL\left(f\right)=N_{t}\left(f\right)+N_{s}\left(f\right)+N_{w}\left(f\right)+N_{th}\left(f\right)$$

where

(4) $$\begin{array}{c} 10\log N_{t}\left(f\right)=17-30\log f\\ 10\log N_{s}\left(f\right)=40+20\left(s-0.5\right)+26\log f-60\log\left(f+0.03\right)\\ 10\log N_{w}\left(f\right)=50+7.5w^{1/2}+20\log f-40\log\left(f+0.4\right)\\ 10\log N_{th}\left(f\right)=-15+20\log f \end{array}$$

Here *s* is the shipping activity factor and k=0.5 for average shipping activity. *w* is the wind velocity in m/s. According to the passive sonar equation [22], the SNR per bit for underwater channel can be described as in Equation (5):

(5) $$\gamma_{b}=SL-TL-NL+DI$$

where SL is the source power level and DI denotes the directivity coefficient. For omnidirectional hydrophones, DI=0. TL is the propagation loss and NL is the noise power spectral density. As we know, the underwater channel suffers severe multipath effects. Here we suppose that the signal mode type is BPSK and the average bit error rate (BER) $P_{b}$ can be obtained according to Rayleigh channel model [25] in Equations (6) and (7):

(6) $$P_{b}=\frac{1}{2}\left(1-\sqrt{\frac{{\bar{\gamma }}_{s}}{1+{\bar{\gamma }}_{s}}}\right)$$

where

(7) $${\bar{\gamma }}_{s}=10^{\gamma_{b}/10}$$

Thus, for a packet with *L* bits, the PER $P_{p}$ can be obtained as in Equation (8):

(8) $$P_{p}=1-{\left(1-P_{b}\right)}^{L}$$

As the nodes in underwater applications always move passively with water currents, the PER between two nodes varies with distance. So we should monitor channel changes and make route decisions based on the real-time $P_{p}$.

### 3.2. Network Model

**Figure 1:**
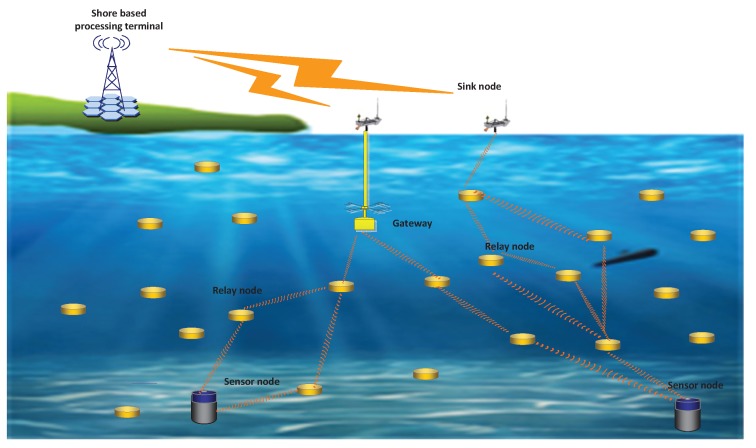
3D network model.

Unlike terrestrial sensor networks, the data transmissions of UWSNs are not limited to 2D region and some applications are aimed at forwarding data packets from seafloor to the sea surface. So the underwater network model is a 3D model as shown in Figure 1. The nodes involved in data transmissions are classified into three categories: the sink nodes, the sensor nodes and the relay nodes. The sink nodes are mainly located on water surface and are responsible for collecting data from the child nodes. Then they forward these data to other gateways or shore-based processing platform through wireless or wired communications. Generally, these sink nodes are equipped with solar panel and have great capacity in data processing. The sensor nodes are equipped with sensors and transmitters. The main functions of sensor nodes are sensing and sampling information obtained from monitored area. The information collected in sensor node is packaged and sent to other nodes. The relay nodes, which are distributed between sink nodes and sensor nodes, are responsible for forwarding data packets to sink nodes. These nodes usually do not have sensors but can decode received data packets and encode data into new packets. The general process of underwater data transmissions is: The sensor nodes collect information from monitored area and generate data packets. These packets are forwarded to chosen relay nodes according to the routing protocols. After multi-hop transmissions, data packets reach the sink nodes and are aggregated or delivered to terrestrial processing platform for further analysis. Most routing protocols define a maximum communication range $R_{max}$, nodes inside $R_{max}$ can communicate with each other and data packets cannot be sent to nodes outside $R_{max}$. Here we redefine a reliable communication range *R*, nodes inside *R* can communicate with each other under low PER (≤10%) but other nodes which are outside *R* can also receive and decode packets successfully under high PER (≥10%). This assumption corresponds to real underwater environment and is helpful for joint decoding process. Underwater nodes might move passively with water currents, so we must take routing maintenance into consideration. We assume that each underwater node knows its location because many underwater applications require node position information. The media access control (MAC) protocol we use here is CW-MAC [26], a MAC protocol which uses a random slot window to transmit data packets if the channel is sensed busy. In this paper, we mainly focus on the design of transport protocol and routing protocol.

### 3.3. Overview of NCHARQ

**Figure 2:**
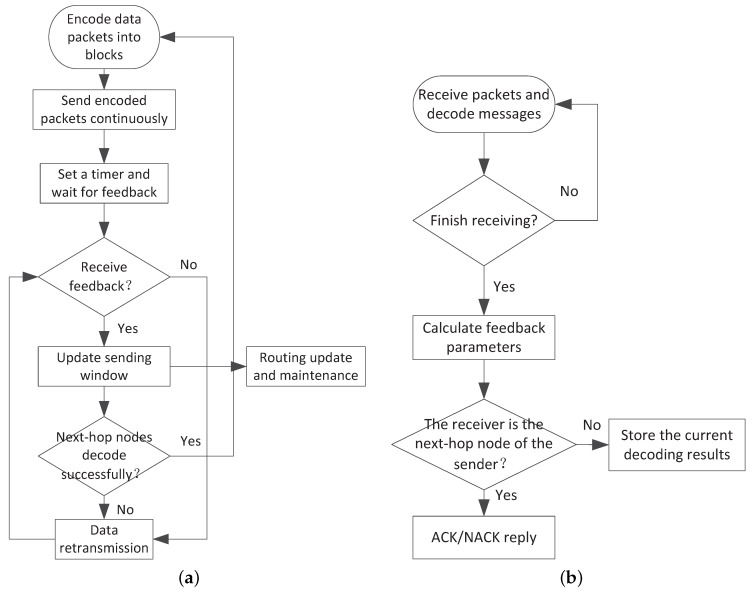
(**a**) The sending procedure for NCHARQ; (**b**) The receiving procedure for NCHARQ.

NCHARQ is a transport protocol which combines network coding and hybrid ARQ techniques [16]. Traditional transfer control protocol (TCP) cannot adapt to underwater environment as TCP requires short round-trip time (RTT) and long RTT is one of the adverse characteristics in underwater environment. The key idea of NCHARQ is to group original data packets into several encoded blocks and data are sent block by block. This improvement can significantly reduce redundant feedback messages. Meanwhile, the transmission power is optimized by controlling the window size of sending block. The flow chart of NCHARQ is given in Figure 2. At the beginning of the sending procedure, the sending node $S_{n}$ sends a block of encoded data packets continuously by multi-casting. The number of packets in one sending block is $W_{i}$ for the *i*th sending procedure. Each encoded packet *m* contains the following information: the unique ID of current sending block $B_{m}$, the coding vector $C_{m}$, the unique ID of next hop node ${Node}_{m}$ and the remaining number of unsent packets $N_{rm}$. $S_{n}$ will wait for feedbacks if all the packets in current block are sent. The neighborhood nodes of node $S_{n}$ receive the encoded packets and try to decode with packets in the same block. When a receiver detects $N_{rm}=0$, it will send back the number of unrecovered packets $N_{fail}$ (Negative acknowledgement, NACK) or the number of redundant packets $N_{rd}$ (Acknowledgement, ACK) according to the decoding result. The sending node $S_{n}$ begins to update the sending block window $W_{i}$ by Equation (9) if it receives the ACK/NACK information.

(9) $$\begin{array}{cc} W_{i+1} & =W_{i}+k_{1}A_{i}+k_{2}\delta_{i}\\ and & A_{i}=\sum_{j=0}^{i}\delta_{i}\\ & \delta_{i}=\left\{\begin{array}{cc} -N_{rd}, & if\;decode\;successfully\\ N_{fail}, & else \end{array}\right. \end{array}$$

Here, $W_{i+1}$ is the updated window size. $A_{i}$ is the accumulated error which reflects the slow change trend of underwater channel and $\delta_{i}$ reflects the instant channel changes. $k_{1},k_{2}$ are impact factors of long time and instant channel changes, respectively. The value of $k_{1},k_{2}$ is determined by the channel change speed and the requirement for loop filter convergence speed. We will give the parameter analysis and numerical simulations in Section 5. Considering that the packet loss is a random event and the packet loss rate of one data send-and-receive procedure cannot reflect the channel quality accurately, we use Equation (9) which is based on loop filter to update the window size $W_{i}$ and thus the effect of packet loss rate estimation fluctuation is reduced. Meanwhile, the window size can converge into a stable state quickly and thus the sending efficiency is improved. Here we will use $W_{i}$ as an important parameter for routing maintenance and this will be further discussed in Section 4. After updating window size, nodes determine to resend data packets or start a new send-and-receive round according to the received feedbacks. If NACKs are received, the unique IDs of unrecovered data packets are extracted from NACK packets and the corresponding data packets are retransmitted. If ACKs are received, nodes can start to send a new block.

NCHARQ can adjust the number of sending packets according to the channel quality and thus optimize the transmission power and reduce the energy consumption. As feedbacks and network coding techniques are included in transmission procedure, NCHARQ can control the packet delivery ratio more efficiently compared with pure forward error correction (FEC) technique. Moreover, the feedback procedure is operated after all the packets in one block are transmitted and this scheme can greatly reduce transmission delay which is brought by long RTT. Meanwhile, the estimation of sending window $W_{i}$ can be used as an important evaluation parameter which can lead to routing update and maintenance. So frequent broadcasting beacon messages are not needed any more and packet collisions are reduced.

## 4. Design of Network Coding Routing Protocol

In this section, we present the detailed design of NCRP, including the protocol design overview, initial routing construction, network coding design and routing update and maintenance.

### 4.1. Protocol Design Overview

**Figure 3:**
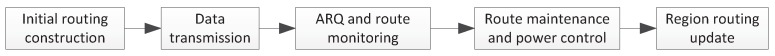
Overview of NCRP.

The overall design of our proposed protocol is presented in Figure 3. Firstly, an initial routing path is constructed. Here we apply receiver-side routing and the destination node is responsible for broadcasting beacon messages. The aim of initial routing construction is not only finding a reliable path to forward data to the destination node, but also including enough auxiliary nodes to assist the data transmission. The detailed design of initial routing construction is given in Section 4.2. At the beginning of data transmission, data packets are encoded with random network coding. Random network coding can ensure that the probability of sending repetitive data packets is low under the premise of lacking information exchange among relay nodes. Nodes can jointly decode messages from all neighborhood nodes. Related encoding and decoding design is given in Section 4.3. The relay nodes jointly decode messages from neighbourhood nodes and determine whether current routing path need to be updated or not. The transmit power is allocated according to the channel condition among nodes. When the transmit power is decreased to zero or increased beyond a predetermined threshold, nodes need to update the routing paths. In order to reduce the effect of partial adverse channel problems to the overall path, we only update the paths near the nodes which have adverse channel problems. This scheme can complete routing update in a short time and have less effect on the overall routing paths. Routing update and maintenance are described in Section 4.4.

### 4.2. Initial Routing Construction

In this part, we will discuss issues about how to find a reliable route to forward data from the source node to the destination node. There are two steps in initial routing construction: The first is to find a primary path which has the minimal hops and shortest distance to the destination. We call nodes in this path the primarynodes. However, nodes in the primary path might have relative long distance between each other, thus the channel condition is poor and the links might have high PER. In order to solve the above problem, we add some secondarynodes into the primary path at the second step. These secondary nodes are distributed between every two primary nodes and are responsible for sending supplemental packets so that the receiver can obtain enough packets for successful decoding. The initialization process is shown in Algorithm 1.
**Algorithm 1** Routing Initialization1:**procedure** Initialize(Node)2:    **if** Node∈DestinationSet **then**3:        Dm(Node)←0;4:    **else**5:        Dm(Node)←∞;6:    **end if**7:    Dn(Node)←∞;8:    nId(Node)←null;9:    rId(Node)←null;10:   Dth←∞;11:**end procedure**

The parameters that each node stores are: Dm (the path distance between current node and the destination node after multi-hop transmission), Dn (the distance between current node and its next-hop primary node), nId (the next-hop node Id in the primary path), nPos (the 3D geographical position of next-hop primary node), rId (the next-hop Id of secondary node), Dth (the sum of distance between next-hop secondary node and its two neighborhood primary nodes). At the beginning of routing protocol, Dm=0 for the destination node and Dm=∞ for others. Other parameters are set as in line 7∼10.

Algorithm 2 is the process of sending and receiving beacon message. Each beacon transmitted message contains three parameters: Dm (the length of primary path), Pos (the geographic position of sending node) and Id (the unique Id of current node). When detecting changes in the primary path, a random time slot timer is set before broadcasting the changed beacon messages (line 1∼8). Suppose node *m* receives beacon messages from node *r* and node *n* is the next hop primary node of node *m*. After receiving beacon messages from node *r*, node *m* calculate DistanceMR and DistanceRN, which are the distance between node *r* and *m* and distance between node *r* and *n*, respectively (line 11∼12). Then node *m* checks the overall path length towards the destination node. If the path length becomes shorter, node *r* can be chosen as a new next hop primary node and node *m* updates its beacon messages and sets a timer to broadcast the changes. If not, we consider whether node *r* can be chosen as a secondary node between node *m* and node *n* or not. Here we choose the node with the minimal DistanceMR+DistanceRN value. This can ensure that the secondary node is close to the line between node *n* and *m*. Moreover, we require that DistanceMR and DistanceRN are both smaller than the distance between node *m* and node *n* (line 21∼23). This requirement can ensure that the secondary node can receive enough packets for decoding. When node *n* cannot decode successfully, the secondary node *r* will send extra encoded packets to node *n* under better channel condition. The beacon messages are sent by the destination node at the beginning. After multi-hop transmission, all the reachable node can find a path to the destination node and the nodes near this path are included into secondary node sets.
**Algorithm 2** Beacon Process1:**procedure** BroadcastBeacon(Node)2:    **if** beacontimeoutexpired **then**3:        packet.addheader(Dm(Node));4:        packet.addheader(Pos(Node));5:        packet.addheader(Id(Node));6:        broadcast packet;7:    **end if**8:**end procedure**9: 10:**procedure** ReceiveBeacon(Node,Packet)11:    DistanceMR←CalculateDistance(Pos(Node),Packet.GetPosition());12:    DistanceRN←CalculateDistance(Packet.GetPosition(),nPos(Node));13:    **if** DistanceMR+Packet.GetDistance()<Dm(Node) **then**14:        Dm(Node)←DistanceMR+Packet.GetDistance();15:        Dn(Node)←DistanceRN;16:        nId(Node)←Packet.GetId();17:        rId(Node)←null;18:        nPos(Node)←Packet.GetPosition();19:        Dth(Node)←∞;20:        Set a timer for BroadcastBeacon(Node);21:    **else if** DistanceMR<Dn(Node)&DistanceRN<Dn(Node)&DistanceMR+DistanceRN<Dth(Node) **then**22:        rId(Node)←Packet.GetId();23:        Dth(Node)←DistanceMR+DistanceRN;24:    **else**25:        drop packet;26:    **end if**27:**end procedure**

It is worthy to be mentioned that the beacon messages in NCRP do not need to be sent frequently as most receiver-side routing protocols do, although the nodes in underwater environment move quickly and the channel might change accordingly. Frequent broadcasting beacon message can solve the node mobility problem but it brings low channel utilization and high energy consumption. Here we combine routing update and maintenance with data transmission, which improves the channel utilization greatly. The related algorithms are present in Section 4.4.

### 4.3. Network Coding Design

Network coding is a new coding scheme which is based on network and multi-node coding. The conventional approach of sending information from one node to multi-node is to send information to relay node equally, and these relay nodes just store and forward the information they receive. This scheme is simple but cannot use the channel resource efficiently. Some relay nodes might send repetitive packets so the receiving nodes have much data redundancy. Network coding is proposed to solve high energy consumption and low data delivery ratio in multi-cast network and it has been attracting attention since it is proposed. In network coding, each relay node recodes data packets before sending them to the next hop node. Based on the multi-cast feature in wireless network, network coding can significantly reduce repetitive data sending, decrease energy consumption and prolong the network lifetime. In UWSNs, data transmissions need more energy and the delay of data retransmission is longer compare with terrestrial wireless network. So network coding can play a greater role in UWSNs.

**Figure 4:**
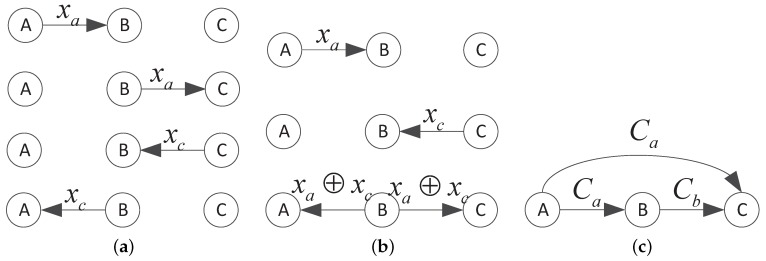
(**a**) The conventional approach of multi-casting; (**b**) The network coding approach of bi-direction transmission; (**c**) The network coding approach for unidirectional transmissions.

Network coding can be illustrated in Figure 4. Node *A* and node *C* wish to send packets $x_{a}$ and $x_{c}$ to each other and node *B* is a relay node which can relay data to node *A* or *C*. In Figure 4a, according to the conventional multi-hop transmission approach, there are four steps in data transmissions: (1) node *A* sends $x_{a}$ to node *B*. (2) node *B* forwards $x_{a}$ to node *C*. (3) node *C* sends $x_{c}$ to node *B*. (4) node *B* forwards $x_{c}$ to node *A*. If node *B* recodes packets using network coding before sending out, the transmission process can be decreased to three steps as shown in Figure 4b: (1) node *A* sends $x_{a}$ to node *B*. (2) node *C* sends $x_{c}$ to node *B*. (3) node *B* broadcast the exclusive-or (XOR) of $x_{a}$ and $x_{c}$ to node *A* and node *C*. Node *A* and *C* can recover the original data packets by XOR with their own sent packets. This simple change can decrease the propagation delay significantly. For unidirectional transmissions, we can illustrate the transmission efficiency improvement by a simple three node transmission case in Figure 4c. We wish that node *A* sends data packets to node *C* and node *B* is the relay node. With the multi-cast effect in UWSNs, the packets sent by node *A* can be received by node *B* and *C* under a certain error probability. As the distance between node *A* and *B* is shorter than the distance between node *A* and node *C*, the quality of underwater channel between node *A* and *B* is better. So node *B* can receive enough encoded packets for successful decoding while node *C* needs more packets. If node *B* recodes the received packets by network coding and sends a few encoded packets to node *C*, node *C* can jointly decode messages successfully with packets from node *A* and *B*. This scheme can reduce the repetitive packets transmission and improve the data transmission efficiency. This advantage is highlighted when there are many nodes (more than 3) participating in data transfer.

From the above analysis we know that the key of designing network coding is to find a proper coding scheme which can avoid repetitive data transmissions. In 2003, Ho et al. proposed random linear network coding [27] which is widely used currently. The key idea of linear network coding is to encode data packets by random linear combination at the sender side, and to decode messages by matrix inversion at the receiver side. The detailed procedure can be described as follows: At the sender side, data packets are grouped into several clusters. Suppose $X_{1},\ldots ,X_{K}$ are data packets in a cluster and $Y_{1},\ldots ,Y_{N}$ are packets after encoding. Then $Y_{i}=\sum_{j=1}^{K}g_{ij}X_{j}$, i=1,…,N. Here $g_{ij}$ are chosen randomly from finite field $F_{2^{q}}$ and $g_{i1},\ldots ,g_{iK}$ are code vectors which are added into the packet header. Generally, *N* is a little larger than *K* to ensure that the receiver can receive enough packets for successful decoding. When the receiver obtains M,M∈(K,N) encoded packets with uncorrelated code vector, the original data packets can be recovered by matrix inversion. This scheme can keep the independence of each code vector from different nods and avoid the repetitive transmissions, but it is not appropriate for UWSNs. The reasons are listed as follows: (1) It is complex to calculate matrix inversion and each decoding round needs the participation of at least *K* encoded packets. The complexity makes it difficult to decode instantly. (2) The sender needs to add the code vector $g_{ij}$ into the packet header and this long code vector might reduce the transmission reliability and increase the transmission power.

Based on the above analysis, we design a simple but efficient network coding scheme which is based on the fountain codes [28]. The fountain codes are a class of erasure-correcting codes. The classic fountain codes are Reed–Solomon codes, Tornado codes, LT codes [28,29,30] and so on. Like random linear network coding, the fountain codes encode *K* data packets into *N* encoded packets, N≥K. Considering the demands for real-time decoding and reducing the packet header length, we design a simple network coding scheme for UWSNs based on LT codes (SLT).

#### 4.3.1. SLT Encoder

Suppose $X_{1},\ldots ,X_{K}$ are data packets in a block. Then the the encoded packet $Y_{i}$ can be obtained in Algorithm 3.
**Algorithm 3** SLT Encoder1:**procedure** SLT_Encoder()2:    $d_{n}\leftarrow GetDegree\left(\rho \left(d\right)\right);$3:    $S_{i}\leftarrow SelectPackets\left(d_{n}\right);$4:    $Y_{i}\leftarrow XOR\left(S_{i}\right);$5:    Return $Y_{i}$;6:**end procedure**

Here the function of GetDegree() is to randomly select a degree $d_{n}$ from degree probability distribution ρ(d). SelectPackets() means to randomly select $d_{n}$ data packets from $X_{1},\ldots ,X_{K}$ and the output encoded packet $Y_{i}$ is the XOR of the *K* packets. Here $d_{n}$ is the number of data packets participated in an output packet. The key problem is the design of degree probability distribution ρ(d). Luby et al. proposed the robust soliton distribution which is used for a large number of data packets. This distribution is not appropriate for UWSNs because the number of sent packets is small and the bit length of code vector is relatively long. To solve the above problems, we design a simplified degree distribution ρ(d) based on the simple variant of tornado (SVT) codes proposed by Xie et al. [31]. In our designed ρ(d), we set the maximum degree $d_{max}$ and the probability of $d_{n}>d_{max}$ equals zero. So each code vector can be expressed as the combination of data packet IDs and the degree $d_{n}$. This operation can reduce the length of code vector significantly. Meanwhile, we need to consider the tradeoff between decoding successful probability and the irrelevance of different packets. An example of degree distribution is presented in Section 5.1 and several degree distributions are also tested for analysis and comparison.

#### 4.3.2. SLT Decoder

We hope that each receiver can decode and reply immediately after receiving an encoded packet. Fortunately, Luby et al. proposed a simple way to solve this problem is by message passing [28]. However, the decoding algorithm proposed by Luby needs to use all the received packets at each decoding round. So we design an improved decoding algorithm for real-time decoding in Algorithm 4. In this decoder, each node stores the set of recovered packets F and the set of unrecovered packets Z. When a new encoded packet $Y_{i}$ is received, the node deletes all the recovered packets from $Y_{i}$ by XOR. Then the node calculates the remaining degree of $Y_{i}$. If the degree equals zero, all the messages in this received packet have already been recovered and this packet is useless for decoding process. If the degree is larger than one, this packet will be included into unrecovered packet set Z. If the degree equals one, the corresponding data packet can be recovered immediately and this packet is included into recovered packet set F. Once a new $Y_{i}$ is added into F, all the packets in Z delete $Y_{i}$ and check if their degrees equal one. If so, new recovered packets are found and the decoding process continues until no one-degree packet is found. This decoding process can delete the useless packets and only needs a few XOR operations. So the speed of decoding process can be improved significantly. It should be pointed out that the start of the decoding process relies on the reception of one-degree packet (or the original data packets). Unless the decoder cannot have enough decoded messages for further decoding. Considering the packet loss in underwater channel, the proportion of one-degree packets in total encoded packets is advised to be over 30% in practical applications. This proportion will be further discussed in Section 5.1. Meanwhile, as SLT is a variant version of fountain codes, the decoding scheme cannot guarantee complete retrieval of the input packets even in perfect channel conditions if the received packets are not enough. However, with a proper degree distribution design, Luby et.al. proved that the decoder can complete decoding successfully with *N* output packets (*N* is a little larger than the number of input packets) under high probability [28]. The analysis of successful decoding probability in our designed degree distribution is also given in Section 5.1.
**Algorithm 4** SLT Decoder1:**procedure** SLT_Decoder($Y_{i}$)2:    $Y_{i}$: new received packet;3:    F: the set of recovered packets;4:    Z: the set of unrecovered packets;5:    $Header\leftarrow RemoveHeader(Y_{i});$6:    $C_{i}\leftarrow GetCodeVector\left(Y_{i}\right);$7:    $Degree\leftarrow GetDegree(C_{i});$8:    **for** $a_{k}\in C_{i}$ **do**9:        **if** $TestRecovered(a_{k})=true$ **then**10:           $Y_{i}\leftarrow xor\left(Y_{i},P_{a_{k}}\right);$11:           Degree←Degree−1;12:        **end if**13:    **end for**14:    **if** degree=1 **then**15:        $F\leftarrow F⋃Y_{i};$16:        **for** $z_{k}\in Z\&\left(z_{k}\;contains\;Y_{i}\right)$ **do**17:           $z_{k}\leftarrow xor\left(z_{k},Y_{i}\right);$18:           $Degree_{z_{k}}\leftarrow Degree_{z_{k}}-1;$19:           **if** $Degree_{z_{k}}=1$ **then**20:               $SLT\_Decoder(Z_{k});$21:           **else if** $Degree_{z_{k}}=0$ **then**22:               Remove $z_{k}$ from Z;23:           **end if**24:        **end for**25:    **else if** Degree=0 **then**26:        drop packet;27:    **else**28:        $Z\leftarrow Z⋃Y_{i};$29:    **end if**30:**end procedure**

During the decoding process, we will calculate two parameters for transport layer feedback. One is $N_{fail}$, which is the unrecovered packet number and the other is $N_{rd}$, which is the number of redundant packets. These two parameters will be sent back to the sender for data retransmissions and power allocation as described in Section 3.3.

### 4.4. Routing Update and Maintenance

The nodes in UWSNs generally moves with water currents because of lacking of fixed platform. The network topology changes heavily over time and the initial route might not be appropriate any more. Based on this, we design a routing update and maintenance scheme which can adapt to channel varying. This scheme can update the inefficient nodes in route paths and have little effect on the overall route and data transmission. From Section 3.3 we know that the sending window size $W_{i}$ can reflect the channel changes and the node contribution to the network. When $W_{i}$ in a certain node is relatively small, this node just sends a few supplementary packets and most needed packets have already been sent by other nodes. So the node contribution to the network is small. When $W_{i}$ in a certain node becomes zero or even negative, there is no need for this node to send packets any more and we should delete this node from the route to save energy and sending time. However, when $W_{i}$ becomes relatively large, it means that the channel between this node and its next hop node is poor and we need to add new relay node to this link to maintain the transmission stability. So we use Equation (10) to update the route.

(10) $$\left\{\begin{array}{cc} W_{i}\leq 0, & delete\;node\\ W_{i}\geq \alpha N_{data}, & add/update\;\;relay \end{array}\right.$$

Here $N_{data}$ is the number of original data packets in a block. α is the redundant factor. Generally, the more nodes contribute in sending packets, the less α is.

**Figure 5:**
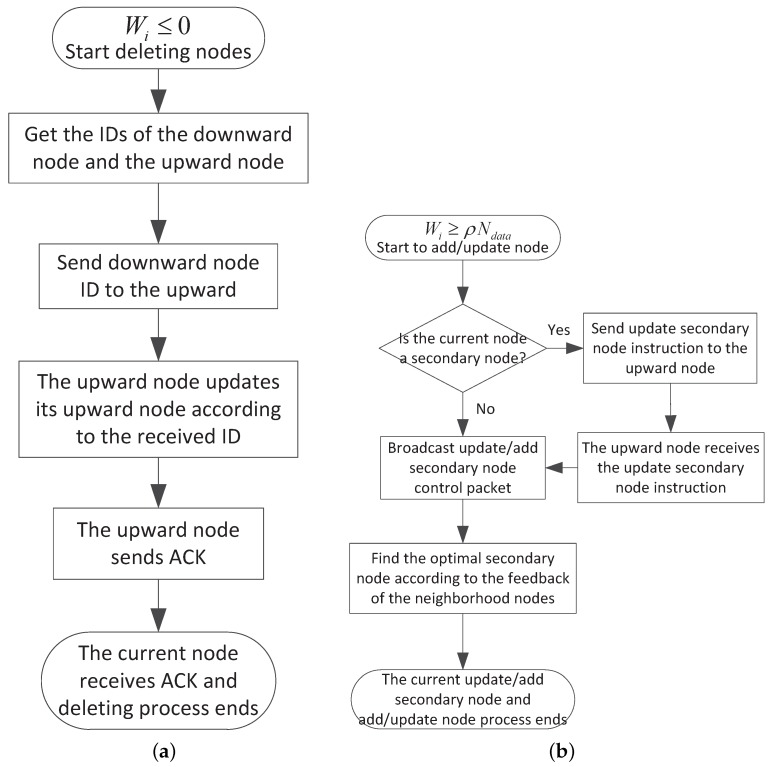
(**a**) The process of deleting nodes; (**b**) The process of adding/updating nodes.

The detailed procedure of route update and maintenance is shown in Figure 5. When detecting $W_{i}\leq 0$, node $S_{n}$ start to delete node as in Figure 5a. Firstly, node $S_{n}$ finds its upward node $S_{n-1}$ according to the received packets. Then node $S_{n}$ obtains its next hop secondary node $S_{nr}$ and next hop primary node $S_{n+1}$ from its stored memory and sends the ID of $S_{nr}$ to node $S_{n-1}$. If node $S_{n}$ does not have a secondary node, $S_{nr}$ can be replaced with $S_{n+1}$. After receiving the deleting control packet, node $S_{n-1}$ replaces its next hop node $S_{n}$ with $S_{nr}$ or $S_{n+1}$. Next, node $S_{n-1}$ sends ACK to inform node $S_{n}$ of the successful deleting operation. Node $S_{n}$ stops sending the deleting control packet if it receives the ACK feedback. When node $S_{n}$ detects $W_{i}\geq \rho N_{data}$, the routing protocol adds a new secondary node into the route or replaces the inefficient node with a better one as shown in Figure 5b. We should check if node $S_{n}$ is a secondary node first. If so, an updating secondary node instruction is sent to its upstream node and the upstream node begins to update its secondary node once it receives the control packet. If not, node $S_{n}$ begins to update the secondary node of itself. The updating process is similar to beacon process in initial route construction in Section 4.2. Node $S_{n}$ broadcast the update requirement packet to its neighborhood nodes. The neighborhood nodes send their geographic position back to node $S_{n}$ after receiving the requirement. Node $S_{n}$ then finds the optimal secondary node according to the criterion in Algorithm 2. The routing update and maintenance algorithm only changes a few nodes in the route and the link still maintains stable in the update process. Moreover, packet collisions can be avoided because only a few nodes send the control packets. Compared with periodic beacons, our protocol can repair the inefficient link in a more timely way.

## 5. Performance Evaluation

In this section, we will conduct simulations and analyze the result compared with the current UWSNs routing protocols VBF, HHVBF, DBR and VAPR. Here we use NS-3 simulation tool, a discrete event simulator [32], to verify the effectiveness and validity of NCRP. The relative simulation parameters are set in Table 1. The underwater MAC protocol we use here is CW-MAC [26], which is a MAC protocol based on a slotted contention window scheme. If the channel is sensed busy, the nodes will choose a random slot and resend packets. In our settings, if two nodes are 400 m apart, the packet loss rate of beacon messages, data packets and feedback packets are 0.019, 0.100, 0.014, respectively. When the two nodes are 1 km apart from each other, the packet loss rate becomes 0.07, 0.40, 0.07. So the reliable transmission range of data packets is about 400 m, but 60% of the data packets can still reach the destination if the transmission range becomes 1 km. In the mobility model, we assume that the nodes mainly move horizontally due to the function of floating bodies and mooring lines. Based on the underwater applications and channel varying, we set 60 data packets in each transmission block. In our simulations, each run lasts 7000 s. Unless otherwise specified, we report the average value of 50 runs.

### 5.1. Effects of Degree Distribution

**Figure 6:**
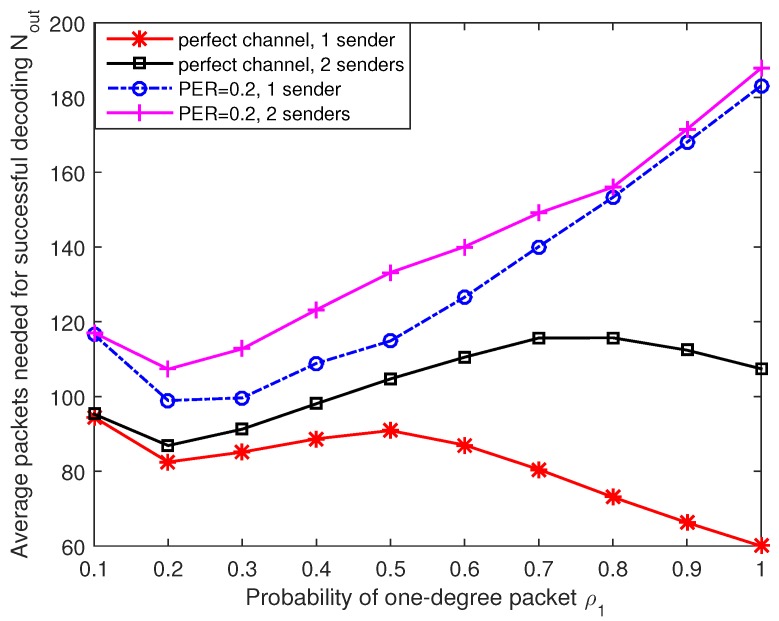
Average number of needed output packets for different probabilities of one-degree packets distribution.

In this part, we discuss the effects of different degree distributions and present a rough optimal degree distribution for our design based on the simulation results. Firstly, we discuss the effects of one-degree packets. The channel models here are considered to be a perfect channel (PER = 0) and an erasure channel (PER = 0.2), respectively. The number of original data packets is set to 60 and we calculate the average value of 500 trials for each test. Figure 6 presents the average number of needed output packets $N_{out}$ under different probabilities of one-degree packets distribution $\rho_{1}$. Here we limit the maximum number of data packets in an output packet $N_{\rho \max}=6$ and then the length of code vector can be shortened to 48 bits. Without loss of generality, other degree probability distributions are set to ρi=1−ρ11−ρ1Nρmax−1Nρmax−1,i=2,…Nρmax. In addition, we define $N_{r\max}=6$ in this simulation, which is the maximum number of times that a data packet can be used in all output encoded packets. When there is only one sender under perfect channel, the average number of needed packets fluctuates smoothly with varying one-degree packet distributions. As we have discussed in Section 4.3.2, the start and continuation of decoding rely on the reception of one-degree packets. If the number of one-degree packets is not enough, the receiver needs more output packets for successful decoding. However, too many one-degree packets may lead to high probability of decoding failure because only a few output packets have overlap between each other. The decoder is inefficient as a large number of received packets have already been recovered by the receiver. This situation is extremely obvious when the channel have a certain packet error rate and $\rho_{1}$ is close to 1. The number of needed output packets is over three times of the original data packets in this case. As we use joint coding/decoding method, we should consider the situation when the received packets come from several independent nodes. The following assumptions are set in the next simulation: There are three nodes involved in data transmissions, one for receiver and the other two are senders. In this two senders case, each sending node sends packets to the receiver with equal probability. From Figure 6 we can see clearly that the performance of two sender case is more sensitive to the number of one-degree packets compared with one sender situation. If the output packets contain too many one-degree packets and the average degree is too low, the sender need to send much more packets so that all the original data packets are chosen at least once in the received encoded packets.

From the above analysis we can see that the decoding performance can be improved with more one-degree packets and higher average degree value. However, the interaction of these two factors constraints the design of degree distribution. In order to further improve the decoding performance, we increase the probability of one-degree packets and high degree packets in the next simulations. Without loss of generality, we simulate and analyze the following six degree distributions.

- Case 1: Degree value = [1 4 6], Degree probability distribution = [0.300 0.175 0.525], Average degree = 4.15, $N_{r\max}$ = 6.
- Case 2: Degree value = [1 4 6], Degree probability distribution = [0.400 0.175 0.425], Average degree = 3.65, $N_{r\max}$ = 6.
- Case 3: Degree value = [1 4 6], Degree probability distribution = [0.500 0.075 0.425], Average degree = 3.35, $N_{r\max}$ = 6.
- Case 4: Degree value = [1 4 6], Degree probability distribution = [0.600 0.175 0.325], Average degree = 3.25, $N_{r\max}$ = 6.
- Case 5: Degree value = [1 2 5 6], Degree probability distribution = [0.300 0.175 0.200 0.325], Average degree = 3.60, $N_{r\max}$ = 6.
- Case 6: Degree value = [1 4 6], Degree probability distribution = [0.500 0.075 0.425], Average degree = 3.35, $N_{r\max}$ = 8.

**Figure 7:**
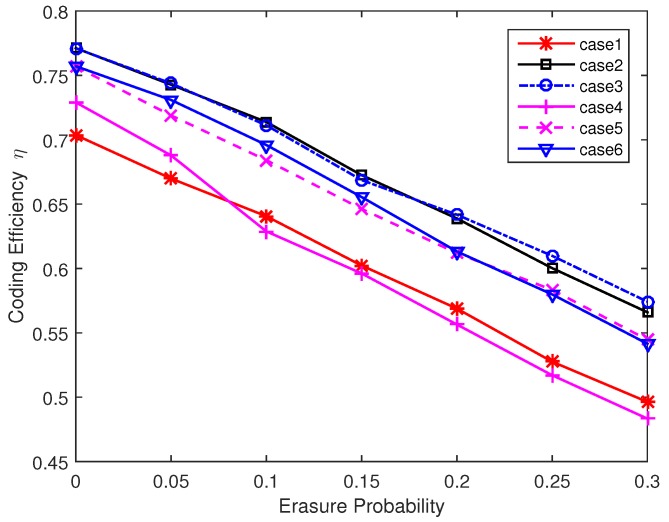
Coding efficiency η for different degree distribution (Two nodes).

**Figure 8:**
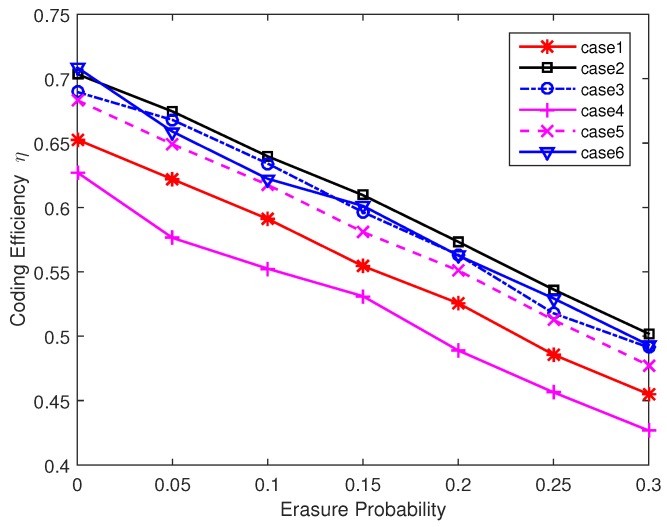
Coding efficiency η for different degree distribution (Three nodes).

**Figure 9:**
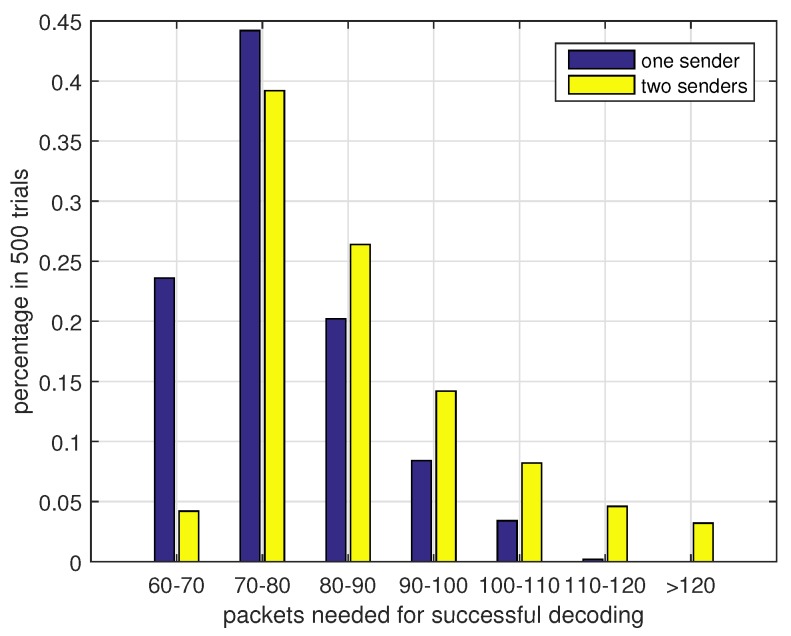
The percentage of packets for successful decoding in 500 trials.

Figure 7 presents the coding efficiency η (the number of original data packets/the number of encoded packets, 0≤η≤1) of different degree distributions in one sender situation. If η is close to 1, it means that the sender only needs to send a few packets for decoding messages successfully. From the results we can see that a relatively low average degree is needed to complete the decoding process. However, if the average degree is too small, data packets cannot be covered effectively and the decoding efficiency reaches a low value. The value of $N_{r\max}$ also affects the coding efficiency. A larger $N_{r\max}$ may lead to a relatively low η because the nonuniform distribution level of data packets selection is higher and some data packets may only have small chances to be included in the output encoded packets. Next we discuss the performance of two sender case. In the two senders case, each sending node sends packets to the receiver with equal probability. Figure 8 presents the results under this assumption. Compared with two nodes data transmission in Figure 7, the three nodes coding scheme is a bit inefficient. This is because the two senders have a certain probability of sending duplicated packets. This probability is higher when we increase the number of low degree encoded packets. From the results we can also see that a large $N_{r\max}$ in case 6 can reduce the duplicated packets sending and improve the coding efficiency in the two senders case. From the Figure 7 and Figure 8 we can conclude that case 3 can work well in both scenarios and we will use case 3 in the following simulations. To further verify the effectiveness of our chosen degree distribution, we present the statistical data of case 3 in perfect channel. The results are shown in Figure 9. From this figure we can see that the decoding process can complete successfully within 100 received packets at a high probability. Although the two sender case performs worse than one sender case, the probability of over 120 encoded packets is less than 5%. So the choice of our degree distribution can satisfy our design. For practical applications, the choice of degree distribution should consider the channel condition and node density. If the channel condition is good and few nodes can be used for auxiliary transmission, we prefer low average degree distribution and vice versa.

### 5.2. Effects of Loop Filter Parameters

In this section, we will discuss the impact of loop filter parameters. The design of loop filter parameters is an important part in our protocol and the convergence rate and stability of loop filter can greatly affect the protocol adaptation to the channel variance. We set a simple four nodes data transmission scenario to verify the adaptation of NCRP. There is one sending node, two relay nodes and a destination node in this simulation. These nodes are distributed in a straight line and the distance between each two neighborhood nodes is 400 m, so the total transmission range is 1200 m. The number of data packets in a block is K=60 and the initial window size $W_{0}=N=80$. We keep the value of *K* unchanged but the window size $W_{i}$ (the output packets *N* in each node for the *i*th block) varies with channel changes. We will analyze the following five cases:

- Case 1: $k_{1}=2^{-7},k_{2}=2^{-2}$.
- Case 2: $k_{1}=2^{-8},k_{2}=2^{-2}$.
- Case 3: $k_{1}=2^{-8},k_{2}=2^{-3}$.
- Case 4: $k_{1}=2^{-6},k_{2}=2^{-3}$.
- Case 5: $k_{1}=2^{-9},k_{2}=2^{-3}$.

**Figure 10:**
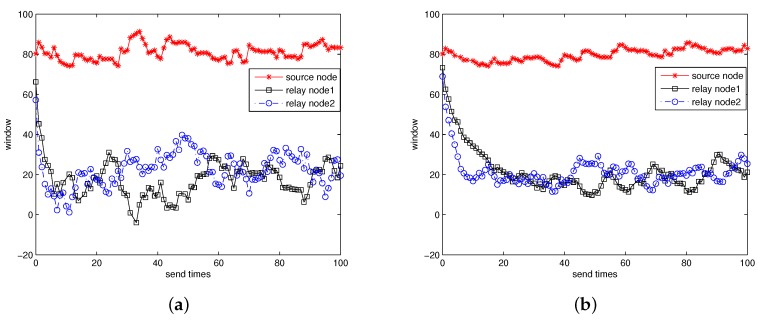
(**a**) The window size estimation for case 1; (**b**) The window size estimation for case 3.

Here $k_{1},k_{2}$ are from Equation (9). Figure 10 shows the window size estimation curve for case 1 and case 3. From the figure we can see that NCRP can obtain the redundant information and complete convergence in a short period of time. However, different nodes may converge to different values. For the source node, it need to send about 80 packets to relay node 1 for successful decoding. This is because there is a certain packet loss rate in the underwater channel and the source node is the only sender for relay node 1. For other nodes, we do not need to send so many packets any more because a large proportion of packets have already been sent by the previous nodes and the receivers only need a few supplementary packets for successful decoding. So the relay nodes only need to send about 20 packets which is much less than that sent by the source node. Figure 10a shows the window estimation curve for case 1 and we can see clearly that the window size estimation can complete convergence very fast (within 10 sending times). However, the value fluctuates fiercely. In addition, Figure 10b shows that the parameter settings of case 3 may extend the time for convergence but the curve seems much more stable than that of case 1.

**Figure 11:**
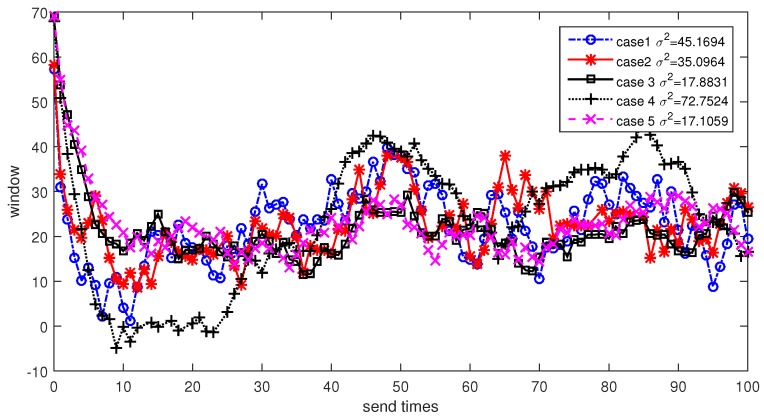
Window size estimation at relay node 2 for different loop filter parameters.

Next we will discuss the performance of different loop filter parameters. For the sake of easy observation, we focus on the convergence curve of relay node 2. We will analyze the convergence rate and variance under different cases. The results are shown in Figure 11. From the figure we can see that case 5 has the minimal variance while the variance in case 4 is the largest. It means that a pair of small $k_{1}$ and $k_{2}$ can reduce the adverse effect of channel noises and lead to a stable window estimation value. However, the convergence speed of case 5 is the lowest which means small $k_{1}$ and $k_{2}$ decrease the effect of feedback value and lead to a long time for loop convergence. Considering the practical requirements in most underwater applications, we prefer case 3 which is a tradeoff between convergence speed and stability.

### 5.3. Performance Analysis

In this part we will analyze the performance of NCRP compared with VBF, HHVBF, DBR and VAPR, and we define the following metrics to measure the performance of SDRT.

1. Packet delivery ratio (PDR): This metric is defined as in Equation (11)
  (11) $$PDR=\frac{Packets\;received\;at\;sink\;nodes}{Packets\;sent\;by\;source\;nodes}$$
2. Average energy tax (AET): AET is defined as the average energy consumption in each node for delivering a packet successfully as in Equation (12).
  (12) $$AET=\frac{Total\;Energy}{{Packets}_{succeed}\times Nodes}$$
3. Average end-to-end delay (EED): EED is defined as the average time it takes for sending a packets from the source node to the destination successfully.

**Figure 12:**
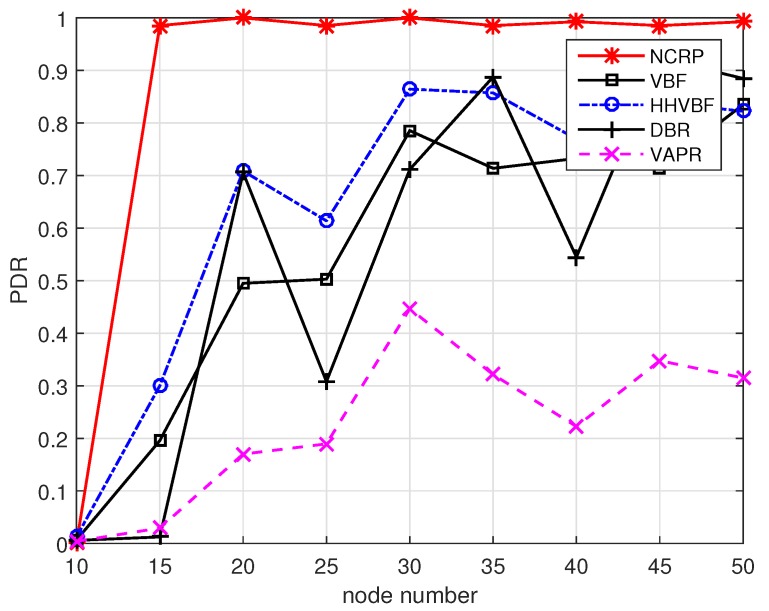
PDR performance with varying node numbers.

According to the parameter settings in Table 1, we compare the performance with node number from 10 to 50. Firstly, the PDR performance is shown in Figure 12. From the figure we can see that it is hard for all routing protocols to find a reliable communication route from the source node to the destination node when node density is relatively low. So the PDRs almost equal zero when node number is 10. If the number of nodes is increased, the PDRs for all protocols increase in various degrees. VBF protocol only uses nodes in a straight pipe line from the source node to the destination node, so it is easily affected by node distribution. If there are no enough nodes in the pipe line, VBF cannot realize efficient data transmissions. HHVBF is an improved version of VBF, which uses multiple pipe lines for transmission and is not limited to the straight line from the source to the destination. When the optimal nodes cannot receive packets successfully, the suboptimal nodes in the transmission range can be chosen for transmission. The PDR of HHVBF can reach 80% when node density is relatively high. DBR protocol is mainly used for vertical transmission. When the number of sink nodes is small and the sink nodes are not located right above the source nodes, the performance of DBR fluctuates considerably. The above three routing protocols share the same problem: the forwarding priority cannot be distinguished clearly if the neighborhood nodes are too close to each other. So if a node cannot obtain suppression forwarding information from its neighbor, multiple nodes might participate in sending the same data packet and their next hop nodes may receive multiple copies of data packets. The sending procedure is then cancelled as duplicated packets are received and this will result in a certain probability of packet loss. VAPR uses periodic beacon messages to find a reliable route. This scheme cannot adapt to node mobility environment efficiently as it cannot update the neighborhood information in time. Meanwhile, frequent broadcasting control messages may lead to packet collisions. In addition, the length of beacon messages is usually much shorter than that of data packets, so data packets cannot transmit as far as beacon messages and the links found by beacon messages are easily broken when they are used for data packet transmissions. NCRP outperforms other protocols in PDR performance. The reasons are listed as follows: (1) We adopt error control method in our system. The nodes in the path can receive feedback from its next hop nodes and retransmissions are required if they sense a failed transmission. (2) We use secondary nodes for transmissions, which can help improve transmission efficiency in high quality channel. (3) The design of loop filter can detect the channel changes and increase the number of sending packets if the channel quality is poor. (4) NCRP can replace the inefficient nodes with good ones promptly. So the PDR performance for NCRP is improved significantly.

**Figure 13:**
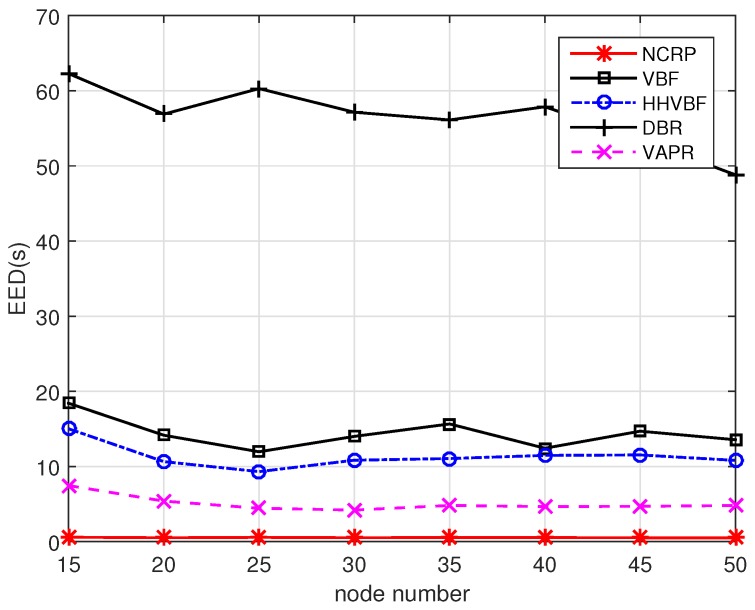
EED performance with varying node numbers.

Next we will discuss the EED performance for different protocols. The results are shown in Figure 13. For the other four protocols, a waiting time before sending procedure is needed to confirm that there are no other nodes sending the same packet. This scheme will result in long end-to-end delay especially when nodes are distributed randomly and most nodes have low priorities for forwarding data. In NCRP, data packets are sent continuously in a block without any waiting time. Moreover, once a block of packets are received successfully, the nodes will send feedback messages to the previous sender and send a new block of packets to its next hop nodes immediately. This scheme can greatly reduce the delay and improve the communication efficiency. The EED in NCRP is only about 10% and 1% of EED in VARP and DBR, respectively.

**Figure 14:**
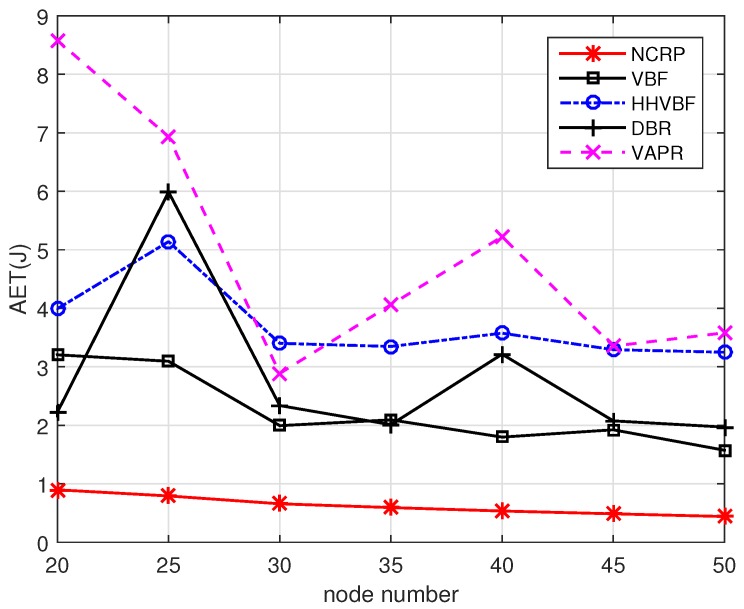
AET performance with varying node numbers.

Figure 14 shows the AET performance in different scenarios. As the power for transmitting data is much larger than that of receiving data, the redundant transmissions will increase the power consumption significantly. For the other four protocols, the sending nodes need to broadcast data packets to their neighborhood nodes and the receiving nodes with high priorities will forward the received packets first. The forwarding procedure in low priority nodes would be suppressed if duplicated packets are received. However, repetitive data transmissions still exist and the reasons are listed as follows: (1) The neighborhood nodes may be too far away to hear each other. (2) The packets sent by high priority nodes are not able to reach their neighborhood nodes due to the high PER in UWSNs. (3) Nodes cannot receive the suppressed information in their waiting time as their priorities are too close. Except for repetitive data transmission, these four protocols cannot use the packets received from unstable link effectively. Compared with other protocols, NCRP does not limit the maximum transmission range for one hop. Nodes can jointly decode messages with packets received from different sending nodes. Meanwhile, the transmission power is optimized as we adopt loop filter and feedbacks in our design. Last but not the least, the inefficient nodes in the routes can be replaced timely and this can reduce the unnecessary energy consumption. So NCRP outperforms other protocols significantly in terms of average energy consumption and prolong the network lifetime. In the 50 nodes scenario, the AET of NCRP is only about 28% and even 12.3% than that of VBF and VARP.

## 6. Conclusions

In this paper, we propose NCRP, a cross-layer routing protocol based on network coding for UWSNs. NCRP takes full use of multi-cast feature in underwater wireless networks and designs an efficient way to find a reliable data transmission link. The process of NCRP can be divided into two parts: initial routing construction and route maintenance. Beacon messages are only used in initial routing construction and route can be updated along with data packets transmissions. The long propagation delay for control messages is then saved and the demand for neighborhood nodes information is also decreased. Meanwhile, as we use network coding method for multiple nodes transmission, NCRP does not limit the transmission range for one hop transmission and the receivers can jointly decode messages with received packets from several neighboring nodes. So data packets received from unstable links are used effectively without any waste. The update of selecting next hop relay is based on the current channel quality and this can make the data transmissions more reliable and avoid the occurrence of void areas. The channel utilization and packet delivery ratio are also improved by real-time transmission rate control and route maintenance. Extensive simulations based on NS-3 show that NCRP outperforms VBF, HHVBF, DBR and VAPR in terms of packet delivery ratio, end to end delay and average energy tax. In future work, we would like to explore the real implementation of NCRP and improve the performance by introducing optimal node deployment technique into the design.

## Figures and Tables

**Table 1:** Simulation Parameters.

Parameter	Value
Data Rate	10 kbps
Center Frequency	12 kHz
Bandwidth	10 kHz
Mode Type	FSK
Packet Error Rate Model	ns3::UanPhyPerNoCode
Signal Noise Model	ns3::UanPhyCalcSinrDefault
Acoustic Propagation Speed	1500 m/s
UAN Propagation Model	ns3::UanPropModelThorp
MAC Model	CWMAC
Mobility Model	RandomWalk2Dmobilitymodel (speed: 2∼4 m/s, directions are choosen randomly)
Energy Model	Acousticmodemenergymodel (TX: 50 W, RX/Idle:158 mW, Sleep:5.8 mW)
Transmission Output Power	147 dB reμ Pa
Required SNR for Signal Acquisition	10 dB reμ Pa
Payload of DATA	64 Bytes
Number of Data Packets in Each Block	60
Deployment Region	3D region of 1.5 × 1.5 × 1 (length × breadth × depth) km^3^
Node Number	10–50, nodes are randomly deployed
Sink Node Position	(1500,1500,0) m
Source Node Position	(0,0,1000) m
